# Peri-infarct edema leads to overestimation of myocardial salvage in late reperfused myocardial infarction

**DOI:** 10.1186/1532-429X-15-S1-P209

**Published:** 2013-01-30

**Authors:** Iacopo Carbone, Marco Francone, Ingo Eitel, Jan Bogaert, Peter Bernhardt, Pier Giorgio Masci, Sven Plein, Matthias G Friedrich

**Affiliations:** 1 Radiological, Onchological and Pathological Sciences, Sapienza, University of Rome, Rome, Italy

## Background

In patients with acute myocardial infarction (AMI) the myocardial salvage index (MSI) quantified by Cardiovascular Magnetic Resonance (CMR) is defined as the volumetric difference of myocardial edema, reflecting the perfusion bed, and the infarcted tissue.

While after a coronary occlusion time of more than 8 hours, relevant myocardial salvage is highly unlikely, previous data however showed myocardial edema surrounding the infarcted tissue after prolonged occlusion times. We therefore speculated that peri-infarct edema can be consistently observed in very late reperfused MI, indicating that edema in late reperfused MI does not necessarily reflect myocardial salvage.

## Methods

In 7 tertiary hospitals, 80 patients who underwent PCI between 8 to 24 hours after presentation with AMI were included in the study. The time to reperfusion (TTR) was correlated with infarct size (IS), extent of myocardial edema and MSI. Standard CMR protocols were used to characterize the extent of myocardial edema, infarction, microvscular obstruction (MO) and the apparent myocardial salvage index. A stratified analysis was conducted considering subjects divided in four quartiles of TTR.

## Results

The mean TTR was 706±238 minutes. The edematous area was consistently larger than the infarct (mean difference 9.8± 8.3%) and the apparent myocardial salvage index was 31.1±20.1%. There was a strong correlation between the area of infarction and the extent of myocardial edema (r=0.84, p<0.0001). TTR was not correlated with the infarct area (p=0.45), the extent of myocardial edema (p=0.17), MO (p=0.65) or the myocardial salvage index (p=0.19).

## Conclusions

In patients with late reperfused myocardial infarction, myocardial edema surrounding the infarcted tissue is a consistent finding. Its extent is not correlated with the time to reperfusion. These data indicate that in late reperfused myocardial infarction: a) Peri-infarct edema is a consistent finding and thus has to be taken into account when assessing myocardial salvage and b) Relevant myocardial salvage appears very unlikely. These findings have significant implications for the quantification of myocardial salvage by CMR and for studies on the "time window" for myocardial revascularization.

## Funding

None

